# 17β-estradiol does not have a direct effect on the function of striatal cholinergic interneurons in adult mice *in vitro*

**DOI:** 10.3389/fendo.2022.993552

**Published:** 2023-01-04

**Authors:** Erzsébet Kövesdi, Ildikó Udvarácz, Angéla Kecskés, Szilárd Szőcs, Szidónia Farkas, Péter Faludi, Tibor Z. Jánosi, István M. Ábrahám, Gergely Kovács

**Affiliations:** ^1^ Institute of Physiology, Medical School, University of Pécs, Pécs, Hungary; ^2^ Centre for Neuroscience, Szentágothai Research Centre, Pécs, Hungary; ^3^ Department of Pharmacology and Pharmacotherapy, Medical School, University of Pécs, Pécs, Hungary

**Keywords:** 17β-estradiol, cholinergic, striatum, RNAscope, estrogen receptor

## Abstract

The striatum is an essential component of the basal ganglia that is involved in motor control, action selection and motor learning. The pathophysiological changes of the striatum are present in several neurological and psychiatric disorder including Parkinson’s and Huntington’s diseases. The striatal cholinergic neurons are the main regulators of striatal microcircuitry. It has been demonstrated that estrogen exerts various effects on neuronal functions in dopaminergic and medium spiny neurons (MSN), however little is known about how the activity of cholinergic interneurons are influenced by estrogens. In this study we examined the acute effect of 17β-estradiol on the function of striatal cholinergic neurons in adult mice *in vitro*. We also tested the effect of estrus cycle and sex on the spontaneous activity of cholinergic interneurons in the striatum. Our RNAscope experiments showed that ERα, ERβ, and GPER1 receptor mRNAs are expressed in some striatal cholinergic neurons at a very low level. In cell-attached patch clamp experiments, we found that a high dose of 17β-estradiol (100 nM) affected the spontaneous firing rate of these neurons only in old males. Our findings did not demonstrate any acute effect of a low concentration of 17β-estradiol (100 pM) or show any association of estrus cycle or sex with the activity of striatal cholinergic neurons. Although estrogen did not induce changes in the intrinsic properties of neurons, indirect effects *via* modulation of the synaptic inputs of striatal cholinergic interneurons cannot be excluded.

## 1 Introduction

Basal ganglia are a group of deep subcortical nuclei in the brain that are essential for motor learning, formation of procedural memory and motor control. The striatum is the major input nucleus of the basal ganglia and is composed of two regions, the dorsal and the ventral striatum. In primates, the caudate nucleus and the putamen form the dorsal striatum, which corresponds to the dorsomedial (DMS) and dorsolateral (DLS) striatum in rodents, respectively. The DMS and the DLS receive inputs from different areas of the cortex, namely, afferents from the prefrontal and the associative cortex reach the DMS, whereas information from the sensorimotor area conveyed to the DLS (1, 2). Based on histochemical identification, the dorsal striatum is composed of two main compartments known as patches (striosomes) and matrix (3). The dorsal striatum is mostly involved in motor learning, action selection, execution and termination. The ventral striatum is composed of the nucleus accumbens and the olfactory tubercle, and is mainly engaged in goal-directed movement and reward-related behavior (2, 4).

Most of the striatal neurons are GABAergic medium spiny neurons (MSN) also known as spiny projection neurons (SPN). They form the sole output of the striatum (direct and indirect pathways). The remaining ~5% of the striatal neurons consist of different classes of aspiny interneurons including parvalbumin-positive, fast-spiking neurons, somatostatin-positive low-threshold spiking neurons, calretinin-positive neurons, and cholinergic interneurons (5).

Cholinergic neurons form a specific population of neurons in the brain that synthesize and release acetylcholine (ACh) as a neurotransmitter. Using specific markers for the intracellular metabolism of acetylcholine such as choline acetyl-transferase (CHAT), acetylcholine esterase (AChE), vesicular acetylcholine transporter (VAChT), or high-affinity choline transporter 1 (ChT1), cholinergic neurons were identified and localized in several discrete brain regions including the striatum (6). Although only ~1% of the striatal neurons are cholinergic interneurons, the highest levels of cholinergic markers are found in the striatum. Despite the fact that the somata of cholinergic interneurons are mostly located in the flanking region of the extrastriosomal matrix compartment of the striatum, cholinergic interneurons regulate and modulate the function of almost all striatal neurons in both striatosomes and the matrix compartment, innervating them with very extensive and massive axonal arborizations (3, 7). The identification of striatal cholinergic neurons (ChINs) is easy, based on the expression of the aforementioned specific neurochemical markers, their distinct morphological appearance (giant aspiny neurons with large (15-50 μm) soma), and unique electrophysiological parameters such as a relatively depolarized resting membrane potential, I_h_ current, prominent afterhyperpolarization and wide action potential (8). In addition, striatal ChINs act as autonomous pacemakers. Several studies suggest that *in vitro*, spontaneously firing ChINs most probably correspond to tonically active neurons (TANs) identified by *in vivo* recordings in the putamen (9). The ChINs express several receptors for different neurotransmitters as they play a central role in the striatal circuitry. They receive significant input from midbrain dopaminergic neurons (D2/D5 receptors), and other striatal ChINs (nAChR and mAChR). They also have extensive glutamatergic innervation from both the cortex and several thalamic nuclei (ionotropic and metabotropic glutamate receptors) and a variety of GABA-ergic inputs (GABA_A_ receptors) (10, 11). On the other hand, besides modulating the activity of GABAergic and glutamatergic striatal afferents, striatal ChINs exert direct postsynaptic effects on MSN activity, which are the main output of the striatum, *via* primarily M1 subtypes of mAChRs (7).

In the nervous system estrogens play a role in sexual differentiation, synaptic plasticity, neuronal differentiation, and neuroprotection. Estrogens also modulate several striatal functions (12–14).

The cellular effects of estrogens are mediated by three different G protein-coupled receptors, namely ERα, ERβ, and G protein-coupled estrogen receptor 1 (GPER1 or GPR30). These receptors could reside in the nucleus (ERα and ERβ) or have an extranuclear localization. Although using *in situ* hybridization, a few groups (15, 16) detected no expression of ERα and ERβ mRNA in the striatum, the majority of previous studies showed that the expression of estrogen receptors in the rodent dorsal striatum was sparse and was weak to moderate even in positive cells (17–22). In addition, recent findings demonstrate that ERα and ERβ expression are high in mouse pups and decreases with time resulting in low or very low expression in adults (23, 24). Finally, ERα and GPER1 were detected in a small proportion of cholinergic interneurons using electron microscopy (17).

Striatal behavior as assessed using locomotor tests showed large differences between male and female animals under resting conditions or after psychostimulant administration (see (13) for review).

Estrogens have a wide variety of genomic and non-genomic effects on the dopaminergic system and MSN neurons in the striatum (see (12–14, 25) for reviews). Striatal cholinergic neurons have a pivotal role in the modulation and function of striatal microcircuitry interacting DA and MSN neurons among many others (10).

Although there is an extensive literature about the effect of estrogens on dopaminergic neurons there is not much information available about estrogens and striatal cholinergic neurons. Therefore, the aim of the present study was to examine how 17β-estradiol and sex affect the spontaneous activity of cholinergic interneurons *in vitro* in the murine dorsal striatum.

## 2 Materials and methods

### 2.1 Animals

All animals (35 transgenic and 6 wild type C57Bl/6) were bred and kept in the temperature- and humidity-controlled animal facility of the Szentágothai Research Center under a 12-hour light/12-hour dark light cycle. The animals used in the experiments were fed with a standard chow and had access to water ad libitum. All experiments were performed on adult mice older than 3 months in accordance with the regulations of the European Community Council Directive and the Animal Welfare Committee of the University of Pécs. To generate ChAT-Cre-tdTomato transgenic mice ChAT-IRES-Cre knock in mice (B6,129S6-*Chat^tm2(cre)Lowl^*/J) and the reporter mouse line B6,129S6-*Gt(ROSA)26Sor^tm9(CAG-tdTomato)Hze^*/J were crossed.

### 2.2 Tissue fixation and slice preparation

Animals anaesthetized with 0.3-0.35 ml of 2.5% Avertin were transcardially perfusion-fixed with 4% paraformaldehyde (PFA) following perfusion with 0.9% physiological saline solution. Brains were removed and postfixed in 4% PFA overnight. Thereafter, samples were cryoprotected by incubating them in TBS (50 mM Tris, 150 mm NaCl, pH 7.4) containing 30% sucrose at +4°C for 8 hours. Next day, 50 μm sagittal sections kept on dry ice were cut for immunofluorescence staining using a sliding microtome (Leica SM2010 R), and the obtained slices were stored in anti-freeze solution (40 mM Na_2_HPO_4,_ 6 mM NaH_2_PO_4_, 20% (v/v) glycerin, and 30% (v/v) ethylene glycol at –20°C until further processing. For RNAscope experiments, 30 μm coronal sections (Bregma +0.14 to +0.4 mm) were prepared from 3-3 male and female wild type C57Bl/6 animals as described above. In some cases, 50 μm sagittal slices were used.

### 2.3 Immunofluorescence and immunohistochemistry (IHC)

For immunofluorescence staining the cryoprotected slices were washed three times in TBS. Next, tissue permeabilization and blocking of non-specific antibody binding was performed by incubating the slices in 10% horse serum and 0.2% Triton X-100 containing TBS solution at room temperature for 2 hours followed by three washes in TBS. Thereafter, the slices were incubated with goat anti-CHAT (antibody registry number: AB 90650) or goat anti-parvalbumin primary antibody (antibody registry number: AB 2650496) at 1:1000 dilution in blocking solution (10% horse serum and 0.05% Triton X-100 containing TBS) at +4°C for 72 hours. Following three washes in TBS, slices were incubated in blocking solution containing donkey anti-goat secondary antibody conjugated to Alexa647 fluorophore (antibody registry number: AB 2340437) at room temperature for two hours. After three consecutive washes in TBS, nuclei were counterstained with Hoechst 33342 at 1:10000 dilution at room temperature for 5 minutes. Following the final three washes in TBS slices were mounted on microscope slides and covered with Prolong GOLD mounting medium.

We also performed NiDAB immunohistochemical staining for cholinergic neurons in some experiments. Here, following three consecutive 10-minute washes with TBS, the endogenous peroxidase activity was blocked by incubating the slices with 1% H_2_O_2_ in 10% methanol at room temperature for 15 minutes. The permeabilization, the blocking and the incubation step with goat anti-CHAT antibody (antibody registry number: AB 90650) was performed as described above. Thereafter, three consecutive washes with TBS were followed by incubation with biotinylated donkey anti-goat secondary antibody (antibody registry number: AB 2340397) diluted at 1:200 in blocking solution at room temperature for 2 hours. To detect the bound secondary antibodies, slices were incubated with avidin/peroxidase complex (Vectastain Elite ABC HRP kit, PK-6100, Vector Laboratories) diluted in blocking solution after three consecutive washes. Finally, NiDAB in 0.1 M acetate buffer was applied to cover the slices and the samples were developed until the desired color reaction could be observed by monitoring it with a brightfield microscope. Termination of development was achieved by rinsing the slides with Tris buffer. After drying the slices on slides, samples were dehydrated with an ascending concentration series of ethanol washes and mounted using DPX mounting medium.

IHC-stained and fluorescence slices were imaged with a Mantra Quantitative pathology workstation, or a Zeiss LSM 710 confocal laser scanning microscope system (Carl Zeiss, Jena, Germany) equipped with violet-diode (405 nm), multiline argon (457–517 nm), and solid-state (543, 561 nm and 633 nm) lasers, respectively. Images were taken with a 20x (N.A. 0.75) objective using ZEN 2.3 imaging software. Post-acquisition image processing was performed in Fiji software.

### 2.4 RNAscope and confocal laser scanning microscopy

In 30 µm thick, paraformaldehyde-fixed coronal brain sections mRNA transcripts of *estrogen receptors* (ERα, Erβ, and GPER1), and *choline acyltransferase* (CHAT), were visualized with a multiplex fluorescence RNAscope *in situ* hybridization assay (Advanced Cell Diagnostics, Newark, CA) (see **Table 1**). Following three consecutive washes in TBS free-floating sections were mounted on Superfrost Plus Gold adhesion slides (Thermo Scientific, 630-1324, VWR). The labeling of the selected transcripts was performed according to the manufacturer’s instructions. Amplification and detection steps for the selected estrogen receptor and CHAT were carried out sequentially. To ensure the specific staining of estrogen receptors transcripts, labeling of these mRNAs was performed before labeling CHAT mRNA. Nuclei were counterstained with Hoechst 33342, and stained sections were mounted with ProLong Diamond Antifade mountant. After 24 hours curing in the mounting medium, slices were sealed with nail polisher. 3-plex negative control probes for mouse tissue were used on two slices each time RNAscope labeling was performed.

**Table 1:** Expression of estrogen receptor mRNAs in striatal cholinergic interneurons.

	Male			Female		
	Total CHAT neurons	ER+ CHAT neurons	% CHAT neurons expressing ER	Total CHAT neurons	ER+ CHAT neurons	% CHAT neurons expressing ER
ERα	286	28	8.71	167	37	21.85
ERβ	231	119	51.52	156	59	37.82
GPER1	184	26	14.13	153	11	7.19

Striatal cholinergic neurons were counted in fluorescently labeled RNAscope slices for each estrogen receptor type obtained from 3 male and 3 female mice. The percentage of the estrogen receptor expressing CHAT+ cells were calculated by dividing the number of ER+ cholinergic neurons with the total number of cholinergic neurons in one slice.

Sections were imaged using a Nikon C2+ confocal laser scanning imaging system in less than one week later. During each imaging session a fluorescence, stitched, large overview image of the whole slice was taken first using a 10x objective (N.A. 0.45). Next, using high magnification objectives (60x or 100x, N.A. 1.4) 12-bit fluorescent images (512 x 512 pixels) were taken at a Nyquist sampling rate. Because the abundance of transcripts for estrogen receptors are low in the striatum, and somata of striatal cholinergic neurons are large, z-scans were carried out for the entire somata of individual cholinergic interneurons with 1 μm interslice distance, and a pinhole size less than one Airy unit. The laser power and the gain of the photomultiplier tube for each channel were set during imaging slices labeled with the 3-plex negative probes. All images were taken using the same imaging parameters during one imaging session. The localization of each imaged striatal cholinergic interneuron was saved on a superimposed, fluorescent overview image.

The image analysis of the obtained z-stacks was performed in Fiji software using the 3D object counter plug-in. The optimal size and intensity thresholds were selected analyzing slides labeled with negative control probes. The expression of estrogen receptors in striatal cholinergic interneurons were scored based on ACD scoring criteria: Score 0 (no expression): 0/cell, Score 1: 1-3 dots/cell, Score 2: 4-9 dots/cell.

### 2.5 Preparation of acute brain slices

ChAT-Cre-tdTomato transgenic mice under deep isoflurane anesthesia were decapitated, and the brain was removed from the skull. 300 µm thick, sagittal brain slices were cut with a vibratome (Leica VT1200s) in an ice-cold NMDG-ACSF solution composed of (in mM) 92 N-methyl-D-glucamine, 2.5 KCl, 30 NaHCO3, 20 HEPES, 25 glucose, 2 thiourea, 5 Na-ascorbate, 3 Na-pyruvate, 0.5 CaCl_2_·2H_2_O, and 10 MgSO_4_·7H_2_O. Upon finishing the cutting procedure, the slices were transferred into a pre-warmed (32°C) recovery vessel filled with NMDG-ACSF bubbled with 5% CO_2_:95% O_2_ gas mixture for 5-10 minutes. Finally, the slices were transferred into a long-term holding chamber filled with HEPES-ACSF solution consisting of (in mM): 92 NaCl, 2.5 KCl, 30 NaHCO3, 20 HEPES, 25 glucose, 2 thiourea, 5 Na-ascorbate, 3 Na-pyruvate, 2 CaCl_2_·2H_2_O, and 2 MgSO_4_·7H_2_O. The HEPES-ACSF holding solution was continuously bubbled with a gas mixture of 5% CO_2_:95% O_2_ and kept at room temperature. Slices were kept in holding solution for an additional one hour to recover. The pH of all solutions was adjusted to 7.4.

### 2.6 Electrophysiology

Electrophysiological experiments were performed on a Nikon Eclipse FN-1 upright microscope. Cells were visualized with infrared differential interference contrast (DIC) optics using a Nikon 40x NIR Apo N2 water dipping objective (N.A. 0.8). Cholinergic neurons expressing tdTomato fluorescent proteins were illuminated with an epifluorescence excitation light source (CoolLED pE-300). Fluorescence signals were detected with an Andor Zyla 5.5 sCMOS camera.

Patch pipettes were pulled from borosilicate glass capillaries with filament (O.D. 1.5 mm, I.D: 1.1 mm) using a Narishige vertical pipette puller. Pipette resistance was between 3-7 MΩ.

In all experiments acute brain slices were constantly superfused with standard artificial cerebrospinal fluid (ACSF) composed of (in mM): 124 NaCl, 2.5 KCl, 24 NaHCO3, 5 HEPES, 12.5 glucose, 2 CaCl_2_·2H_2_O, and 2 MgSO_4_·7H_2_O with a pH adjusted to 7.4 and bubbled with a 95% O_2_/5% CO_2_ gas mixture. Experiments were carried out at 32°C. All drugs were applied into the bath solution *via* superfusion at least for 5 minutes. 17β-estradiol was dissolved in absolute ethanol to obtain 10 mM stock solution. 17β-estradiol stock solution was diluted 1:100000 to reach 100 nM concentration, and further diluted 1:1000 to obtain 100 pM concentration.

In loose patch or cell-attached patch experiments patch pipettes were filled with the standard ACSF. The liquid junction potential was around zero because the composition of the solutions in the bath and the pipette was the same. Measurements were mostly carried out in current clamp mode using 0 mA holding current. In some tight cell-attached experiment recordings were made in voltage clamp mode using a command potential resulting in zero current passing across the patch. Under these conditions the spontaneous firing pattern is not affected (26). Signals were low pass filtered with 4kHz Bessel filter and digitized at 50 kHz (Digidata 1550B, Molecular Devices).

Offline data analysis was carried out using Clampfit 10.7 software (Molecular Devices). The average frequency of the neuronal action potential firing and the local variation of the interspike intervals over 5 minutes periods were. The local variation (27) was defined as:


$$L_{v}=\;\frac{3}{n-1}\sum_{i=1}^{n-1}{\left(\frac{I_{i}-\;I_{i+1}}{I_{i}+\;I_{i+1}}\right)}^{2}$$

As compared to the coefficient of variation, the local variation is a better firing metric, because it is insensitive to firing rate fluctuations and represents the instantaneous variability of interspike intervals more closely (28).

In cell-attached experiments with a pipette-cell seal resistance over 1 GΩ, the resting membrane potential could be recorded in current-clamp mode (26, 29).

### 2.7 Determination of estrous cycles by vaginal smear

Vaginal smears were taken from female mice by application of 100 µl of physiological saline solution into the vagina followed by aspiration of the flushed fluids. Samples were immediately placed and smeared on glass microscope slides and allowed to dry at room temperature. Dried smears were stained with methylene blue solution for 1 min and washed in tap water. The estrus state was determined using a light microscope with 10x objective (30).

### 2.8 Statistical analysis

For data analysis and graph generation Microsoft Excel 2018 and GraphPad Prism 8 software were used. Data are represented in figures either as sample median ± range or as individual data points. The normal distribution of the sample data was tested with the Shapiro-Wilk test. The obtained average firing rate and the local variation data of control and estrogen-treated groups were compared with Wilcoxon matched-pairs signed rank t-test. The comparison of non-paired experimental data was tested with Kolgomorov-Smirnov test (comparing two groups) or non-parametric Kruskal-Wallis test (comparing several groups). The sample size was based on reports in related literature and was not predetermined by calculation.

## 3 Results

### 3.1 Expression pattern of tdTomato fluorescent protein in the dorsal striatum of ChAT-Cre-tdTomato animals

**Figure 1:**
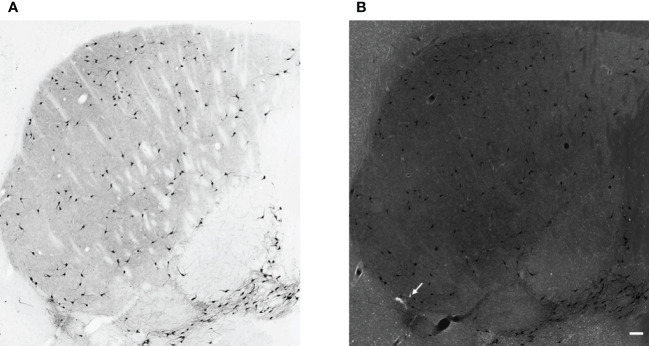
Cholinergic neurons in the striatum. Representative images of DAB immunohistochemistry for CHAT in the striatum from adult female ChAT-Cre-tdTomato transgenic mouse (Panel **A**, brightfield image, and Panel **(B)**, fluorescence image). In panel **B** the white cell represents non-cholinergic but tdTomato-positive cells (white arrow), whereas cholinergic, CHAT-positive interneurons are black in both panels. 10x magnification, scale bar represents 100 μm.

**Figure 2:**
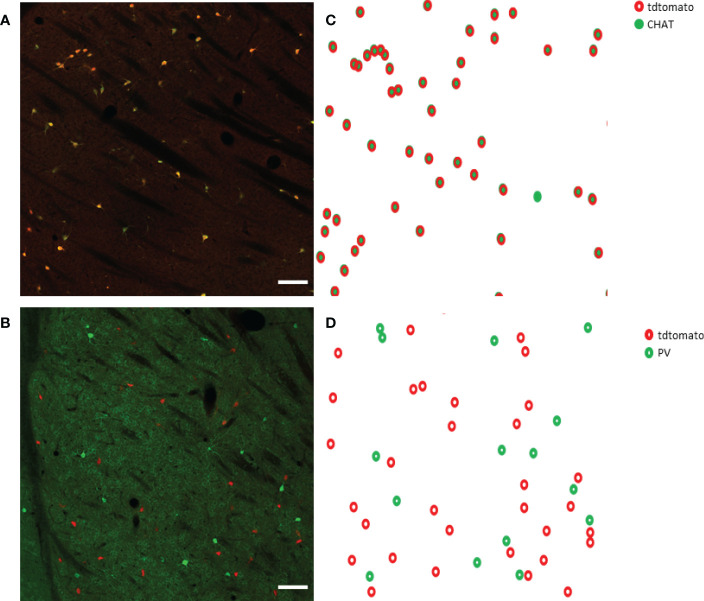
CHAT and parvalbumin (PV) staining in the striatum from a ChAT-Cre-tdTomato transgenic adult female mouse. Representative images show CHAT- and PV-positive cells in green in a sagittal section of the striatum in Panels **(A, B)** respectively. TdTomato-expressing cells are presented in red. CHAT+ cells are marked with filled circles in Panel **(C)**, while open green circles represent PV+ cells in Panel **(D)**. TdTomato-expressing cells are marked with open red circles in both Panels **(C, D)**. 20x magnification, scale bar presents 100 μm.

First, we tested how many cells have ectopic expression tdTomato fluorescence protein in the dorsal striatum. The immunohistochemical staining for CHAT protein showed the well-known morphological characteristics of the giant, aspiny cholinergic interneurons (**Figure 1A**). As depicted in **Figure 1B**, the fluorescence image of the immunohistochemical staining revealed that only a negligible fraction of the tdTomato-positive cells was CHAT-negative. The only cell found to be non-cholinergic is marked with an arrow in **Figure 1B**. The morphology and expression pattern of the fluorescent, tdTomato-expressing cells in the fluorescent image of the dorsal striatum clearly resemble striatal cholinergic interneuron cells (**Supplementary 1**). Furthermore, immunofluorescent labeling of the CHAT protein showed almost complete colocalization whereas absolutely no colocalization was observed between parvalbumin- and CHAT-positive neurons (**Figure 2**). Our data showed that 97.72% of the tdTomato-expressing cells were cholinergic interneurons (927 of 939 cells n = 5 animals).

### 3.2 Expression of ERα, ERβ, and GPER1 mRNA in the cholinergic interneurons of the dorsal striatum

**Figure 3:**
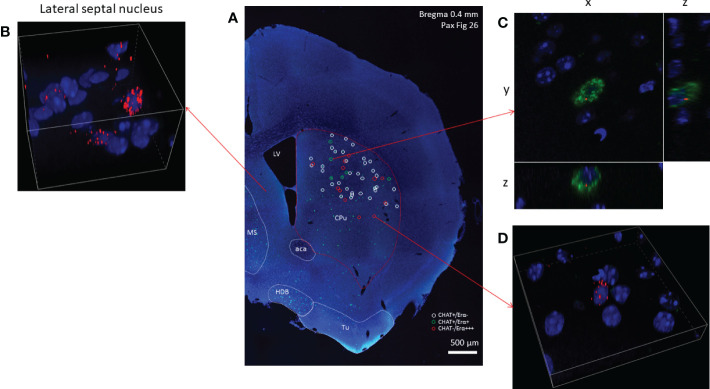
Estrogen receptor alpha mRNA expression in striatal cholinergic neurons. Overview image of the right side of a coronal section obtained from an adult male mouse brain is presented in Panel **(A)** (10x). Green fluorescence labeling show cholinergic cells expressing CHAT mRNA (Panel **A**). White and green circles mark ERα-negative and ERα-positive striatal cholinergic neurons, respectively. Non-cholinergic cells with strong ERα positivity are highlighted with red circles. Cells expressing ERα at high level in the lateral septum are depicted in Panel **(B)** (60x). A representative ERα-positive striatal cholinergic neuron is shown in Panel **(C)** (blue: nuclei, green: CHAT mRNA, red: ERα mRNA, 60x). In Panel **(D)**, one non-cholinergic cell with abundant expression of ERα mRNA is presented (60x). CPu, caudate-putamen; aca, anterior limb of anterior commissure; MS, medial septum; HDB, horizontal limb of the diagonal band of Broca; Tu, olfactory tubercle; LV, lateral ventricle.

**Figure 4:**
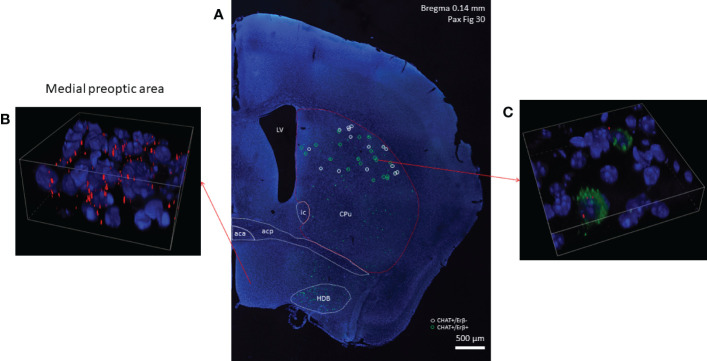
Estrogen receptor beta mRNA expression in striatal cholinergic neurons. CHAT mRNA-positive cells showing green fluorescence labeling in the right side of a coronal section are depicted in Panel **(A)** (10x). In the dorsal striatum white and green circles mark ERβ-negative and ERβ-positive cholinergic interneurons, respectively. Cells with high ERβ mRNA expression in the medial preoptic area are presented in Panel **(B)** Representative confocal image shows the expression of ERβ mRNA in 2 cholinergic interneurons in the dorsal striatum (blue: nuclei, green: CHAT mRNA, red: ERα mRNA, 60x) (Panel **C**). CPu, caudate-putamen; aca, anterior limb of anterior commissure; acp, posterior limb of anterior commissure; HDB, horizontal limb of the diagonal band of Broca; LV, lateral ventricle.

**Figure 5:**
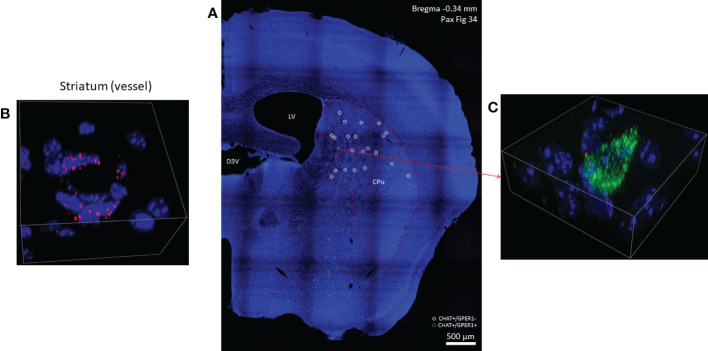
GPER1 (GPR30) mRNA expression in striatal cholinergic neurons. In one half of a coronal section cholinergic neurons marked with green, fluorescent probes used against CHAT mRNA are depicted in Panel **(A)** (10x). GPER1-positive striatal cholinergic interneurons are marked with green open circles while white open circles show cholinergic neurons with no GPER1 mRNA expression in the dorsal striatum. In Panel **(B)**, endothelial cells of a small vessel abundantly expressing GPER1 mRNA are depicted (60x). One GPER1-positive striatal cholinergic interneuron is shown in Panel **(C)** (blue: nuclei, green: CHAT mRNA, red: ERα mRNA, 60x). CPu, caudate-putamen; LV, lateral ventricle; D3V, third ventricle.

Using RNAscope *in situ* hybridization we found that many cholinergic neurons express no detectable ERα mRNA (**Figure 3**) in either sex. A smaller fraction of cholinergic interneurons (8.71% in males, and 21.85% in females), showed weak ERα positivity (**Table 1**). We have to note that there were some non-cholinergic cells that expressed a moderate amount of ERα mRNA (**Figure 3**). Weak ERβ mRNA expression was detected in 51.52% and 37.82% of cholinergic interneurons in males and females, respectively (**Figure 4** and **Table 1**). The plasma membrane estrogen receptor (GPER1) mRNA was found in small amounts in some cholinergic interneurons in both sexes (**Figure 5** and **Table 1**).

### 3.3 Effect of sex and age on spontaneous firing of cholinergic interneurons in the dorsal striatum in male and female mice

**Figure 6:**
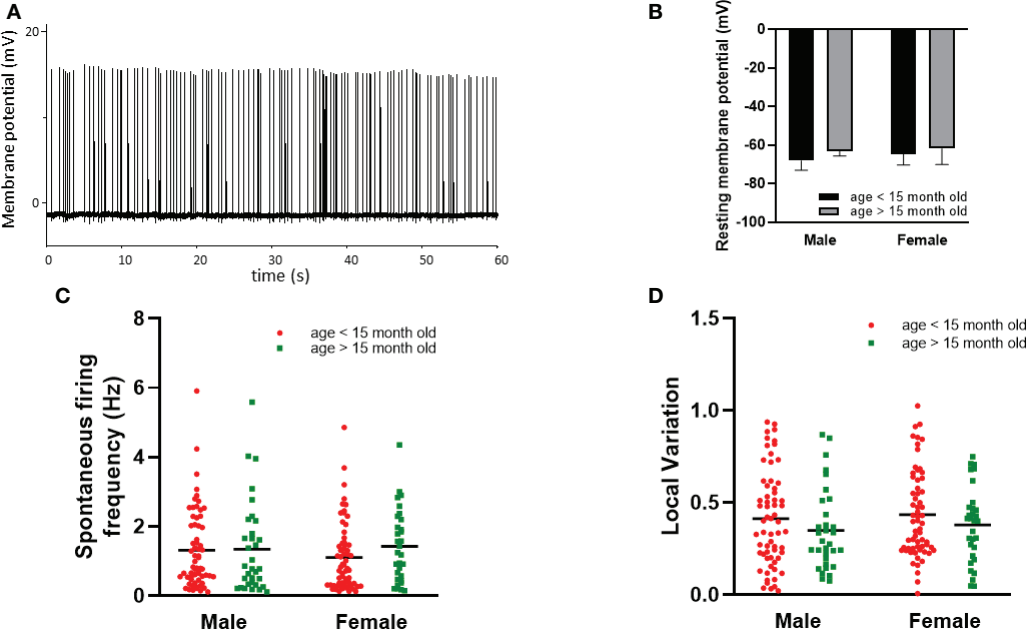
Effect of age and sex on intrinsic properties of striatal cholinergic interneurons. Representative, loose patch recording of spontaneous activity obtained from a striatal cholinergic neuron is depicted in Panel **(A)**. The resting membrane potential, the frequency, and the local variation of spontaneous firing activity in males and females is presented in Panels **(B-D)**, respectively. Data in one gender were further divided based on age.

**Figure 7:**
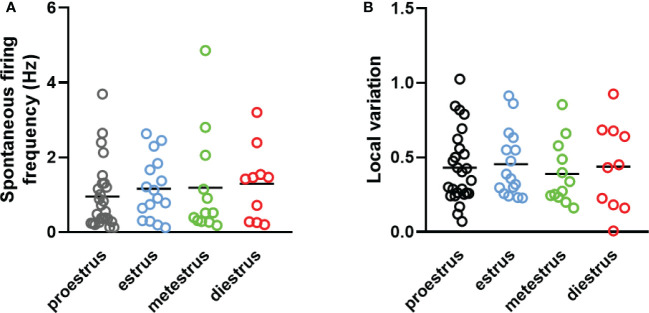
The effect of estrous cycle on spontaneous firing of striatal cholinergic interneurons in adult female mice. The frequency and the local variation of spontaneous activity in cholinergic interneurons in different phases of estrous cycle are presented in Panels **(A, B)**, respectively.

Dorsal striatal cholinergic interneurons are pacemaker cells that are able to fire spontaneously in the absence of any synaptic input (**Figure 6A**). The rate and variation of the spontaneous firing of these interneurons were measured between 10 and 15 minutes after a seal was established in order not to confound the results due to mechanical disturbance of seal formation. Nearly half of the patched neurons showed spontaneous activity at higher than 0.1% Hz frequency in both sexes (97/215 in males, 94/191 in females, respectively).We found no difference in the number of spontaneous active cells between sexes in old animals (age > 15 month), as 54.39% (31/57) of the patched striatal cholinergic neurons were active in females, and 54.10% (33/61) in males. The resting membrane potential measured in cells monitored in cell-attached mode (seal resistance is greater than 1 GΩ) in 61, 58, 12 and 10 neurons obtained from adult male and female, as well as old male and female animals, respectively. No significant difference was found among the different groups (-68.31 mV ± 4.90 in adult males vs -64.72 mV ± 5.63 in adult females, -63.05 mV ± 2.68 in old males vs -61.84 mV ± 8.26 in old females) (**Figure 6B**). We also did not observe any difference in frequency between sexes or detect any effect of age (1.26 Hz ± 1.09 in adult males vs 1.11 Hz ± 1.00 in adult females, p = 0.6754, 1.34 Hz ± 1.33 in old males vs 1.42 ± 1.04 in old females, p = 0.9984) (**Figure 6C**). In addition, local variation in spontaneous firing was not affected by either sex or age (0.43 ± 0.27 in adult males vs 0.44 ± 0.24 in adult females, p = 0.957, 0.382 ± 029 in old males vs 0.38 ± 0.20 in old females, p = 0.958) (**Figure 6D**). In female mice, both frequency and local variation were unaffected by the phase of estrous cycle (**Figures 7A, B**).

### 3.4 Rapid effect of 17β-estradiol on spontaneous firing activity of cholinergic interneurons

**Figure 8:**
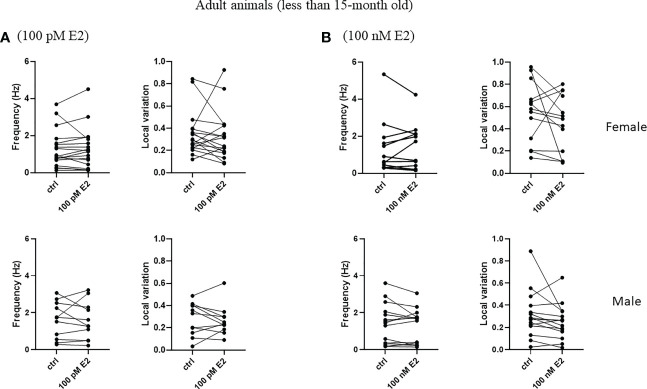
Rapid effect of 17β-estradiol on spontaneous activity of cholinergic interneurons in adult animals. The acute effect of 100 pM or 100 nM 17β-estradiol on spontaneous activity of cholinergic interneurons are shown in Panels **(A, B)**, respectively.

**Figure 9:**
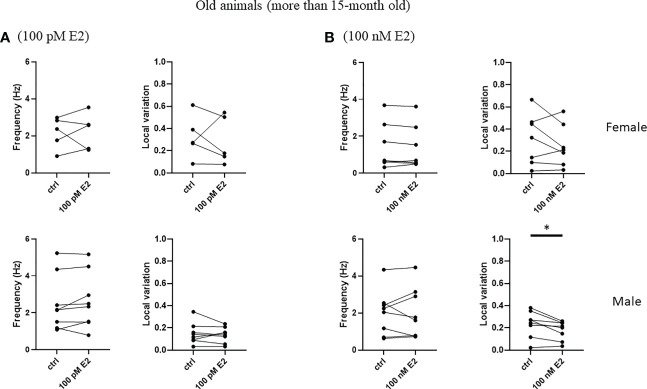
Rapid effect of 17β-estradiol on spontaneous activity of cholinergic interneurons in old animals.The effect of 100 pM or 100 nM 17β-estradiol in 5 minutes on spontaneous activity of cholinergic interneurons are shown in Panels **(A, B)**, respectively. *p< 0.05.

To test the rapid effect of 17β-estradiol superfused into bath, we measured the frequency and the local variation of the spontaneous firing of striatal cholinergic neurons over the first 5 minutes after the administration of 17β-estradiol. 17β-estradiol at 100 pM concentration did not affect neither frequency nor the local variation of spontaneous firing in any of the examined groups (**Figures 8A**, **9A**). In addition, 100 nM 17β-estradiol had no effect on local variation in adult or old females (**Figures 8B**, **9B**). Interestingly, when we compared the adult (younger than 15 month) with the old (older than 15 month) male animals, 100 nM 17β-estradiol significantly lowered the local variation only in the old animals (from 0.235 ± 0.118 to 0.178 ± 0.085, n = 8, p = 0.0184) but not in the adult animals (from 0.316 ± 0.210 to 0.252 ± 0.157, n = 15, p = 0.1326).

## 4 Discussion

Sex and gonadal hormones can influence many neural functions to a large extent in different brain regions. Estrogens evoke two kinds of effect that are different in many ways. The rapid effects can be evoked in seconds or minutes, and they do not activate the transcription of any target genes. The underlying mechanisms are non-genomic and mediated by extranuclear, mostly membrane-bound estrogen receptors such as ERα and GPER1. Various intracellular signaling pathways including phosphatidylinositol 3-kinase (PI3K)/Akt pathway, mitogen-activated protein kinase (MAPK)/extracellular regulated kinase (ERK) pathway, protein kinase A, and protein kinase C pathways are involved in rapid, non-genomic effects (31). Because estrogens are synthesized also in the brain and modulate many neuronal and glial cellular functions *via* non-genomic effects, they are considered as neurosteroids (32). In contrast, the genomic effects develop in hours to days, but they are long lasting because changes in gene transcription and protein synthesis are involved. The genomic effects of estrogens are mediated by the nuclear estrogen receptors ERα and ERβ. Estrogens form a complex with estrogen receptors and that complex binds to the estrogen response element (ERE) in the promoter region of the target genes resulting in the modulation of the transcriptional activity.

It is well documented that there are large sex-related differences in nigrostriatal and mesolimbic dopaminergic pathways (12, 13, 25). Ligand binding studies of striatal D1 and D2 dopaminergic receptors that indicate changes in expression and/or binding affinity showed clear sex differences in rodents (12, 13, 33). Studies performed on ovariectomized female rats showed that ovariectomy decreased both D1 and D2 ligand binding, which was prevented by administration of 17β-estradiol (33–37). Administration of 17β-estradiol increased the binding to striatal D1 receptors in male mice after 6 days, but not at earlier timepoints after the treatment (38, 39). In contrast, binding to D2 receptors were decreased in both male and female mice after 24 hours (38). In non-human primates, D2 receptor availability was reported to be higher in the luteal phase as compared to the follicular phase, and the number of D1-D2 heteromeric complex expressing neurons and the density of D1-D2 complexes were higher in females (40, 41). Besides the changes in dopamine receptor function by estrogens in the striatum, dopamine turnover is also greatly affected by estradiol. The expression of the dopamine transporter (DAT) was lower in males than females and the level of DAT was dependent on estrous cycle phase and greatly reduced by ovariectomy (40, 42–45).

Estrous cycle phases were also clearly associated with the level of extracellular dopamine concentrations in the striatum (highest in proestrus lowest in metestrus/diestrus). In addition, 17β-estradiol rapidly enhanced K^+^- or amphetamine-induced dopamine release in the striatum suggesting underlying non-genomic mechanisms (see (12, 25) for reviews).

Dopaminergic input has a large effect on striatal cholinergic neurons. The predominant, D2-mediated, inhibitory effect is achieved by modulating the I_h_ current and enhancing the slow inactivation of voltage-gated Na^+^ channels. The synaptic input is reduced by inhibition of high-voltage-activated Ca^2+^channels. Dopamine enhances ACh release from striatal cholinergic neurons by promoting the opening of non-selective cation channels and the closure of K^+^ channels (see (7, 10) for reviews).

A rapid decrease in L-type calcium current and cAMP responsive-element-binding protein (CREB) phosphorylation induced by 17β-estradiol *via* estrogen receptor alpha (ERα), estrogen receptor beta (ERβ) and mGluR was demonstrated in striatal MSN (46, 47).

Therefore, the main goal of the present study was to examine the rapid effect of 17β-estradiol and the influence of sex on the spontaneous activity of striatal cholinergic interneurons. First, we examined the expression of estrogen receptors on cholinergic interneurons in the dorsal striatum. Because of the lack of specific antibodies, we performed RNAscope *in situ* hybridization to detect estrogen receptor mRNA in CHAT-positive cells. Our data showed that subpopulations of cholinergic interneurons express at least one of the estrogen receptors at low levels. In addition, we found sex differences in estrogen receptor-positive populations of cholinergic interneurons. Here, we also observed some non-cholinergic cells that strongly express ERα mRNA. These data are in accordance with previously published data demonstrating the expression of estrogen receptors at low level in the dorsal striatum (17, 20–22). In addition, using electron microscopy, Almey et al. reported that ERα and GPER1 protein labeling is associated with axons and terminals of striatal cholinergic neurons (17). Furthermore, GABAergic medium spiny neurons, which innervate cholinergic interneurons, also express estrogen receptors (18). These data suggest that either directly or indirectly through MSN afferents, estrogens could modulate the activity of cholinergic interneurons.

To test this hypothesis we measured two parameters, namely frequency and local variation of spontaneous activity of cholinergic interneurons. We found that none of these parameters were affected by sex or the phase of the estrous cycle *in vitro* under resting conditions. There was also no difference in resting membrane potential between males and females. These data suggest that the locally produced endogenous estrogens do not have any influence on basal pacemaker activity of cholinergic interneurons. However, the mRNA expression of CHAT, the enzyme that synthesizes acetylcholine, fluctuates during the course of the estrous cycle in different regions of rat basal forebrain including the striatum (48). It was also reported that CHAT mRNA significantly increased in response to OVX (48). Although mRNA abundance might not correlate with the protein abundance, the basal acetylcholine release can be different between sexes and can be dependent on estrogen levels at the same spontaneous firing rate.

We also investigated whether 17β-estradiol can alter the spontaneous activity of cholinergic interneurons in a rapid, non-genomic way. We used 17β-estradiol at two different concentrations, namely 100 pM and 100 nM, that was used before in neuronal patch-clamp studies to investigate the rapid, non-genomic effect of 17β-estradiol on neuronal activity (49–52). Administration of 100 pM, the so called “physiological concentration” of 17β-estradiol, did not influence the frequency or the local variation of basal firing of cholinergic neurons in either sex. In addition, a large “pharmacological 100 nM dose” of 17β-estradiol did not induce any changes in females in 5 minutes. However, we found that while in adult animals there was only a tendency for a decrease in the local variation of spontaneous firing activity of ChINs induced by 100 nM 17β-estradiol, in old animals it was clearly demonstratable. It should be noted that the physiological concentration of endogenous 17β-estradiol in the brain is still not known, so the physiological and the pharmacological concentrations refers to blood levels.

The information encoded in neuronal firing can occur in two ways: in the rate (rate coding) or in the temporal distribution (temporal coding) of spiking activity (53). The rapid effect of large dose of 17β-estradiol on the variation of spiking activity in males suggest that estrogen can rapidly modulate the striatal output *via* MSN activity by altering the regulatory function of cholinergic interneurons. The interpretation of this finding in the context of locomotor responses in rodents needs further investigation. Nevertheless, our data are consistent with blocking the production of endogenous estrogens, as aromatase inhibition did not alter the firing pattern discharge, the current-voltage relationship parameter, or the EPSC amplitude of cholinergic interneurons in male rats (54). On the other hand, long-term potentiation (LTP) induced by a high-frequency stimulation protocol was completely prevented by aromatase inhibition which was restored by the dopamine receptor 1 (D1R) agonist SKF-82958 (54). In addition, the increase in striatal acetylcholine level induced by the dopamine agonist apomorphine was significantly attenuated by moxestrol, a potent estrogen (55). These data suggest that cholinergic activity can be modulated by 17β-estradiol indirectly *via* dopaminergic afferents under certain circumstances.

In summary, we found that sex has no effect on basal activity of striatal cholinergic neurons, while a rapid, non-genomic effect of 17β-estradiol at a pharmacological dose was observed on firing variability only in old males. Our data suggest that underlying mechanisms of sex differences in striatal behavior does not include differences in basal intrinsic electrophysiological properties of striatal cholinergic neurons. However, the possibility that E2 regulates ChINs indirectly *via* acting on its afferent cannot be excluded.

## Data availability statement

The raw data supporting the conclusions of this article will be made available by the authors, without undue reservation.

## Ethics statement

The animal study was approved by the Local Animal Care Committee of the University of Pécs (BAI/35/51-141/2016 University of Pécs, Hungary).

## Author contributions

EK: design of the study, writing, data analysis. IU: perfusion, sectioning, immunohistochemistry, RNAscope *in situ* hybridization. AK: RNAscope *in situ* hybridization, validation, data analysis. SS: bright-field imaging, image analysis, data analysis and interpretation. SF: data analysis and interpretation. PF: RNAscope *in situ* hybridization, bright-field imaging, confocal imaging, data analysis and interpretation. TJ: data analysis. IÁ: conceptualization, funding acquisition. GK: design and supervision of the study, writing, critical reading, editing, and revising the manuscript. All authors were involved in the critical revision of the manuscript. All authors read and approved the final manuscript.

## Supplementary material

Supplementary 1 TdTomato-expressing cells in a sagittal section of the striatum from adult ChAT-Cre-tdTomato mouse. Red fluorescent cells representing striatal cholinergic interneurons are shown in Panel **A**. Nuclear counterstaining and merged image are presented in Panels **B** and **C**, respectively. 10x magnification, scale bar presents 100 μm.
