# Supplementary figures and images for: 17β-estradiol does not have a direct effect on the function of striatal cholinergic interneurons in adult mice in vitro
Source: Front Endocrinol (Lausanne). 2023 Jan 4;13:993552. doi: 10.3389/fendo.2022.993552 (PMC9848397; doi:10.3389/fendo.2022.993552)

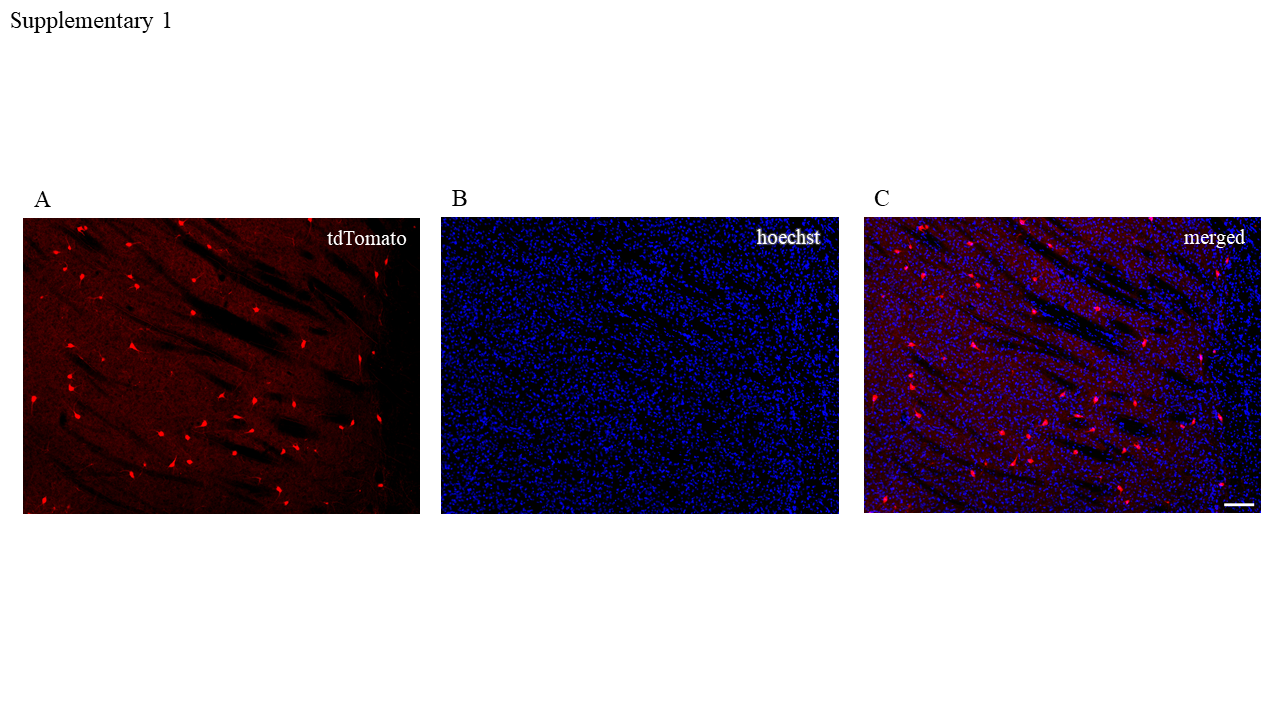

Supplement: Supplementary 1 — TdTomato-expressing cells in a sagittal section of the striatum from adult ChAT-Cre-tdTomato mouse. Red fluorescent cells representing striatal cholinergic interneurons are shown in Panel A. Nuclear counterstaining and merged image are presented in Panels B and C, respectively. 10x magnification, scale bar presents 100 μm. [file Image_1.tif]
